# The Survivorship Bias in Congenital Diaphragmatic Hernia

**DOI:** 10.3390/children9020218

**Published:** 2022-02-06

**Authors:** Emrah Aydin, Nilhan Torlak, Beth Haberman, Foong-Yen Lim, Jose L. Peiro

**Affiliations:** 1 Division of Pediatric General and Thoracic Surgery, Cincinnati Children’s Hospital Medical Center, Cincinnati, OH 45229, USA; foong.yen.lim@cchmc.org (F.-Y.L.); jose.peiro@cchmc.org (J.L.P.); 2 Department of Pediatric Surgery, Tekirdağ Namık Kemal University School of Medicine, 59030 Tekirdağ, Turkey; 3 Cellular and Molecular Medicine, Koç University Graduate School of Health Sciences, 34450 Istanbul, Turkey; ntorlak19@ku.edu.tr; 4 Division of Neonatology, Perinatal Institute, Cincinnati Children’s Hospital Medical Center, Cincinnati, OH 45229, USA; beth.haberman@cchmc.org; 5 College of Medicine, University of Cincinnati, Cincinnati, OH 45267, USA

**Keywords:** congenital diaphragmatic hernia, mortality, LHR, O/E LHR, O/E TLV, postnatal operation

## Abstract

Current literature for congenital diaphragmatic hernia (CDH) focuses on the comparison of the overall mortality in CDH patients. Only a few studies concentrate on analyzing the unstable patients who could not achieve surgical repair, as well as those who could but did not survive after. Hence, this study aimed to analyze the effects of various parameters on the timing of death. A retrospective analysis was performed by using the data of all CDH patients from 2003 to 2016 at a single tertiary center. Patients who were diagnosed with left-sided CDH and expired were included in the study regardless of the cause. Of the 66 expired patients, 5 were excluded due to right-sided CDH. The study population constituted a total of 61 patients, of which 31 patients expired prior to CDH repair, and 30 patients expired at different times after CDH repair. Multinomial regression analysis identified that the ECMO need (B = 20.257, *p* = 0.000, OR: 62.756, 95% CI 10.600–371.384) and O/E LHR (B = 20.376, *p* = 0.000, OR: 70.663, 95% CI 48.716–102.415) values were the independent predictors that influenced mortality in this cohort. Prenatal pulmonary measurements are the major predictors determining the severity of the disease in patients with CDH.

## 1. Introduction

It is always the most acceptable thing to learn from the successful, but this comes with a bounce-back effect because many researchers do not take the survivorship bias into account [1,2]. Survivorship bias is defined as the logical error of concentrating on the patients that could survive and overlooking those that could not, which ends up with some misleading conclusions in various ways [3]. As a result, data based on this selection bias result in deceptive decisions. In other words, just the survivors who outperformed the rest are chosen, and their properties are determined without considering the entire dataset, including those with identical traits who did not do as well.

Congenital diaphragmatic hernia (CDH) occurs due to an embryological failure during the diaphragm development and is seen in 1 in 2500 live births [4,5,6]. While the exact etiology is still unknown, the main pathophysiological consequences are regarded as immature lungs and pulmonary hypoplasia, persistent pulmonary hypertension of neonates, and anomalies in the pulmonary vasculature [7,8,9]. Despite the fact that antenatal diagnosis via ultrasonography (USG), assessment of prognostic variables, prenatal counseling, and therapeutic modalities to the fetus can be used to predict disease outcomes, CDH death and morbidity rates remain high. For the management of the disease and its complications, as well as the management of concomitant malformations, CDH newborns must be hospitalized for an extended amount of time, with long-term follow-up [10,11].

In the last decades, surgical repair of CDH, which is usually not an emergency procedure, has advanced as surgical techniques and treatment strategies have improved [12,13]. Surgical techniques are determined according to the defect size and the patient’s condition, as well as the surgeon’s preferences. Primary repair is predominantly preferred for small defects, while larger defects require a replacement either with a synthetic patch or a muscle flap [14]. These techniques can also be performed when the patient is on ECMO support without significant additional complications [14].

In light of these considerations, most studies in the literature compare patients who survive versus those who do not. However, there is a knowledge gap in the sub-analysis of individuals who died and were unable to receive surgical repair, as well as those who could but did not live despite surgical intervention. Consequently, the goal of this study is to investigate the influence of numerous parameters on the timing of death.

## 2. Materials and Methods

After receiving ethics committee permission (IRB #2017-6361), the investigations were carried out in accordance with the 1975 Declaration of Helsinki, which was amended in 2013. Prior to being included in the study, all participants gave their consent during their admission to the center. Between 2003 and 2016, all data were evaluated retrospectively in a single tertiary hospital with a fetal center. The data containing patient demographics, associated problems, preoperative parameters, surgeries, postoperative results, and survival were collected using an institutional database and hospital records. Parental surveys and the patients’ electronic medical records were used to collect long-term data. Regardless of the initial intervention, surveys were conducted at a particular time. Carrying out additional phone calls to non-responders resulted in a 92 percent inclusion rate. The study included all patients who had left-sided CDH and had passed away, regardless of the etiology. The study population was divided into two groups: Group 1 (patients who died before CDH repair) and Group 2 (patients who died after CDH repair). The primary goal was survival prior to CDH repair, with the reason of death as a secondary outcome.

For statistical analysis, IBM SPSS Statistics 26.0.0 (Chicago, IL, USA) was used. The study sample’s characteristics were reported using descriptive statistics, which showed dichotomous or ordinal data as percentages and continuous data as means with standard deviations. For the demonstration of normal distribution, the Kolmogorov–Smirnov test was performed. For homogeneity of the variables, one-way ANOVA was used; for parametric data, Student’s T-test and Pearson correlation were used; for non-parametric data, Mann–Whitney U, Wilcoxon, and Kruskal–Wallis tests and Spearman correlation were employed. If the *p*-value was less than 0.05, statistical relationships were considered significant.

## 3. Results

We assessed 263 CDH patients prenatally between 2003 and 2016. Among them, 69.2 percent (182/263) were hospitalized for postnatal care. During the same time period, 39 more CDH patients were admitted for postnatal care. As a result, a total of 221 patients were examined. At discharge, the overall survival rate was 73.8 percent (163/221), and the overall survival rate to date was 70.1 percent (155/221). A hernia sac was found in 13.8 percent of patients, 59 percent had liver herniation, and 25.1 percent had a related condition. Additionally, 36.3 percent required ECMO, while 84.3 percent required surgical repair. Five of the 66 patients who died had right-sided CDH; thus, they were omitted from the research. Therefore, the study population constituted a total of 61 patients with left-sided CDH, of which 31 patients expired prior to CDH repair, and 30 patients expired after CDH repair.

In the study population, the average age of mothers during pregnancy was 26.49 ± 6.29 years, and 59% (36/61) of patients were male. The mean gestational age during prenatal diagnosis was 25.41 ± 3.92 weeks. The mean age at delivery was 36.75 ± 2.60 weeks with a mean weight of 2615 ± 700 grams and a mean APGAR score of 3.67 ± 2.56 at the first minute of life and 5.86 ± 2.43 at the fifth minute. The mean length of survival was 100.41 ± 305.28 days (range 1 to 2182). The mean LHR at mid-gestation was 1.03 ± 0.38, while the mean O/E LHR was 26.07 ± 9.41%, and the mean O/E TLV was 10.47 ± 5.86%, where the mean gestational age for the USG was 24.45 ± 2.96 weeks. While only one patient could be shown to have a hernia sac, 40 (65.6%) patients had liver herniation into the thorax. Among all, 37 (60.7%) patients did not have any accompanying disease or malformation. Of those who had comorbidity, the majority were cardiovascular (*n* = 17) and chromosomal (*n* = 6).

The comparison of patient characteristics in each group did not reveal significant differences. The only statistically significant differences were with gestational age and weight at delivery, patient age at the time of expiration, and the presence of a hernia sac (Table 1). There was no statistically significant difference between groups with accompanying disorders, prenatal diagnosis, and maternal age. Fewer patients cannulated with ECMO in Group 1, which, however, did not reach statistical significance. In general, the pulmonary measurements were worse in Group 2 when compared with Group 1.

Univariate analysis showed that the presence of prenatal diagnosis, the gestational age at diagnosis (the earliest the worst), the need for ECMO cannulation, the LHR, and O/E LHR levels were the most critical parameters that determined the death of the CDH patients. A stepwise logistic regression model was performed to assess the multivariate validity in predicting survival. Multinomial regression analysis identified the need for ECMO cannulation (B = 20.257, *p* = 0.000, OR: 62.756, 95% CI 10.600–371.384), and O/E LHR (B = 20.376, *p* = 0.000, OR: 70.663, 95% CI 48.716–102.415) values were the independent predictors that influenced the survival.

The sub-analysis of the patients cannulated with ECMO revealed that 18 out of 42 still could not succeed surgical repair. Among 24 patients that were repaired, one patient was repaired prior to ECMO cannulation, seven patients were repaired after ECMO decannulation, and 16 were repaired on ECMO. Among the 42 expired patients that were ECMO cannulated, 18 expired on ECMO, and 24 expired after decannulation. When comparing both groups, 12/18 expired on ECMO in Group 1 while 6/24 expired in Group 2 (*p* = 0.008, OR = 6.00, 95% CI 1.560–23.072).

Of the 61 patients that expired, 6 expired after they were discharged from the initial hospitalization at a mean age at death of 692.00 ± 793.80 days (range 35–2182). One patient died from cardiac arrest during laparotomy for intestinal volvulus, and another patient died from cerebral edema and herniation. Of the remaining four patients, parents declined an autopsy. Pulmonary underdevelopment was accepted as the leading cause of death in one of these four patients. Among the 55 patients that expired prior to discharge, the parents of 25 declined an autopsy. In four patients, it was the parents’ decision to withdraw life-sustaining support. Among the non-CDH causes, one patient expired due to septicemia, one patient had an intraventricular hemorrhage, and one patient expired due to severe congenital heart disease. The autopsies of the rest of the patients, when available, revealed hypoplastic lungs being the major cause of death.

## 4. Discussion

There are numerous studies in the literature that evaluate patients who survive CDH and compare them to those who do not [15,16]. However, some of the patients that expire also differ in their characteristics. Although the treatment for CDH is personalized, certain management strategies still apply to all patients. Regardless of the efforts, some patients could not succeed surgical repair, while some succeeded but expired just post-operatively. For improving their overall survival, it is necessary to comprehend the diversity of these individuals’ characteristics. As a result, the goal of this research was to find parameters that can help distinguish between patients who will succeed with diaphragmatic repair and those who will not.

Advancements in surgical interventions, neonatal care, and treatment strategies have improved CDH management in the last decades. Although these advancements improve patient survival, certain factors, such as the presence of liver herniation and associated defects, reduce the chances of survival [10]. In a previous study by our team, we showed that as more liver mass herniated into the thoracic cavity, ECMO need was increased, and survival of the patients was decreased; moreover, this herniation decreased the prenatal lung measurements as well [10]. Another study investigated the liver position and lung-to-head ratio (LHR) to predict the requirement for ECMO cannulation and survival in isolated left CDH; its results showed that fetuses with liver up and liver down had 45 percent and 93 percent overall survival rates, respectively [17]. The current study supports the literature that proves the association of ECMO need and decreased O/E LHR levels with these patients’ survival. However, no statistically significant difference could be shown with the liver position.

Treatment strategies are based on the position of the hernia and prognostic indicators, which are the hernia sac, herniated liver, and comorbidities [14]. One of these features, liver herniation, affects the need for thoracostomy tube, duration of oxygen requirement, and ECMO; therefore, it is associated with longer lengths of hospital stay. It has been reported that 100% survival can be achieved in patients with isolated CDH without liver herniation. On the other hand, there is a 45–74% decrease in survival rates when the liver is up in the thorax [17,18]. While the presence of a hernia sac formed by the parietal peritoneum and lung pleura has a good effect on prognosis, CDH associated with anomalies has lower survival rates compared to isolated CDH [19]. Even though 38% of live births with CDH were found to have associated anomalies, Cannon et al. reported that 62% of their CDH patients with accompanying anomalies did not survive [20]. In a study focusing on CDH patients in West of Scotland, neural tube defects are regarded as the largest group of major anomalies related to CDH, followed by cardiac anomalies, including Fallot’s tetralogy and ventricular septal defects [21]. In accordance with all findings, the fetus should be hemodynamically stable before the operation. Moreover, even with the improvements in surgical interventions and treatment strategies leading to an increase in survival rates, morbidity and extrapulmonary complications such as recurrence of the hernia, pulmonary hypoplasia, neurodevelopmental delay, and musculoskeletal abnormalities can affect the long-term outcome of severe CDH cases [22,23].

According to Schwartz et al., left ventricular mass can be utilized as a predictor of lung weight and thus, of the need for ECMO and overall survival [21]. Many prenatal criteria to evaluate the severity of the condition rely on pulmonary volumes; the most common measurements are LHR, a standardized version of it, observed-to-expected LHR (O/E LHR), or total lung volumes (TLV). LHR is determined by USG measurements, whereas TLV data are obtained using magnetic resonance imaging (MRI) [24]. The rationale behind the use of O/E LHR is to make an indirect evaluation of the unaffected lung volume and hence the probability of lung hypoplasia [18]. The requirement for ECMO cannulation is primarily dictated by the patient’s prenatal lung measurements and postnatal clinical results. Consequently, all of these are believed to be related to one another. In the current study, however, the lung morphometric parameters in Group 1 were slightly better than those in Group 2, though there was no statistically significant difference. This raised concerns regarding the timing of the surgery and the patient’s preparatory care. Traditionally, CDH repair has not been considered an emergency procedure. Prior to surgery, the patient’s hemodynamics should be stable. However, per the findings in this study, patients who expired prior to surgery had better pulmonary characteristics but could not achieve surgical repair. One can speculate that the patients with worse prenatal pulmonary measurements had more aggressive management, and therefore, they at least survived the surgery. One influencing factor can be that in recent years, the trend has been to repair the estimated worse cases in the first days on ECMO, while several years ago, surgeons mostly performed late repairs or preferred to wait for decannulation from ECMO to perform the diaphragm repair.

There are certain limitations of the study. First, it was a retrospective cross-sectional review that had a constitutional bias on the selection criteria of the patients per timing of repair. Another limitation was the difficulty in evaluating the effect of comorbidities due to the study population being small.

## 5. Conclusions

In patients with congenital diaphragmatic hernia, prenatal pulmonary measurements are the most important indicators of disease severity and mortality. On the other hand, the management method should be aggressive enough to enhance patient outcomes. Despite using all postnatal treatment techniques in a level IV advanced NICU, lung hypoplasia, and thus the inability to wean from ECMO, remains the leading cause of death in this patient group.

## Figures and Tables

**Table 1:** Patient and hernia characteristics, operative and outcome data per group.

	Group 1 (*n* = 31)	Group 2 (*n* = 30)	*p*
Patient characteristics			
Gestational age at diagnosis (weeks)	25.28 ± 4.06	25.54 ± 3.85	0.807
Gestational age at delivery (weeks)	35.99 ± 3.22	37.53 ± 1.44	0.020
Weight at delivery (grams)	2413 ± 749	2825 ± 587	0.020
Maternal age (years)	26.74 ± 6.37	26.23 ± 6.31	0.755
Prenatal diagnosis	28 (90.3%)	29 (96.7%)	0.317
Diagnosed syndrome	7 (22.6%)	7 (23.3%)	0.944
Diagnosed chromosomal abnormality	3 (9.7%)	3 (10%)	0.966
Diagnosed congenital heart disease	10 (32.3%)	8 (26.7%)	0.632
Isolated CDH	21 (67.7%)	16 (53.3%)	0.249
Male	19 (61.3%)	17 (56.7%)	0.714
Patient age at the time of expiration (days)	19.94 ± 21.13	18.67 ± 420.96	0.029
ECMO need	18 (58.1%)	24 (80%)	0.064
Apgar score			
1 min	3.42 ± 2.69	3.89 ± 2.47	0.509
5 min	5.43 ± 2.59	6.21 ± 2.27	0.257
Length of hospital stay (days)	16.65 ± 21.05	75.76 ± 69.88	0.000
Hernia characteristics			
Presence of hernia sac	0	1 (3.3%)	0.000
Liver up	18 (58.1%)	22 (73.3%)	0.747
LHR	1.07 ± 0.42	0.99 ± 0.34	0.482
O/E LHR	27.58 ± 10.83	24.55 ± 7.68	0.258
<15%	1 (3.2%)	1 (3.3%)	
16–25%	13 (41.9%)	15 (50%)	
26–35%	6 (19.4%)	8 (26.7%)	
36–45%	1 (3.2%)	1 (3.3%)	
>45%	3 (9.7%)	0	

Values expressed as means ± standard deviations or counts (percentage of the group). CDH, congenital diaphragmatic hernia. LHR indicates lung-to-head ratio; O/E LHR, observed to expected lung-to-head ratio; ECMO, extracorporeal membrane oxygenation; *p* refers to the measure of the probability that an observed difference could have occurred just by random chance.

## Data Availability

There are no available data to be shared.
